# Man and the Locomotive

**Published:** 1874-12

**Authors:** 


### MAN AND THE LOCOMOTIVE.

Both are machines, both derive their power from the combustion, the burning-up of certain substances—vegetable and animal substances—which have grown. Both are made up of a multitude of different parts of wheels within wheels. If one of these wheels move, all move ; if one is broken, all are deranged in their action, and the wheel mechanism stops.

There are points of difference. The locomotive wears out, and must be repaired by external help. The human machine also wears away. It constantly consumes itself, yet as constantly makes its own repairs, as fast as they are needed, so that the wear is never noticed. A prisoner was bound to the stone floor of a dungeon for many years ; his clanking chain allowed him to make only three or four steps back and forth, and whenever the soft foot fell on the hard rock it wore away the adamant in the deep hollows, and yet the foot of flesh had not worn away a particle. The locomotive must have the heads and intelligence of others to supply it with suitable food. Man has an instinct which craves what he wants, and an intelligence which provides, selects, and prepares the materials which furnish the fuel he needs, and the hands to convey it to himself.

The engineer who knows his business selects such fuel as will best answer his purpose, and is most economical, aiming for that which gives the greatest amount of heat from the smallest quantity, and not forgetting to use that which does the least injury to his machine. Men eat without regard to cost or suitableness ; they eat what they like best, or what they can most easily get. The engineer has a pride in running his machine economically and safely without regard to others. Many men provide for their tables, largely guided by their neighbors, who may have different tastes or greater pecuniary ability, priding themselves on setting as "good a table" as anybody else. Our study ought to be to eat that kind of food which is best for us, which gives the most appropriate nourishment at the least cost. This is true economy—the economy of intelligence. The time will come, sooner or later, in the history of this country, when a systematic and wise attention must be paid to considerations of this character ; and all might be benefited now, both as to health and purse, by carefully studying this subject.

